# The Role of Triglycerides in the Development of Atherosclerosis

**DOI:** 10.15388/Amed.2026.33.1.4

**Published:** 2026-07-22

**Authors:** Vaida Cerebiejūtė, Živilė Girkantaitė, Egidija Rinkūnienė, Silvija Gimžauskaitė, Jolita Badarienė

**Affiliations:** 1 Vilnius University Faculty of Medicine, Vilnius, Lithuania; 2 Cardiology and Angiology Center, Vilnius University Hospital Santaros Klinikos, Vilnius, Lithuania; 3 Vilnius University Faculty of Medicine, Vilnius, Lithuania; 4 Vilnius University Faculty of Medicine, Vilnius, Lithuania; 5 Vilnius University Faculty of Medicine, Vilnius, Lithuania

**Keywords:** triglycerides, triglyceride-rich lipoproteins, remnant cholesterol, residual risk, atherosclerosis, trigliceridai, trigliceridų turtingi lipoproteinai, remnantinis cholesterolis, liekamoji rizika, aterosklerozė

## Abstract

**Background:**

Atherosclerotic cardiovascular disease (ASCVD) is a chronic inflammatory disease of the arteries. The primary driver of atherosclerosis is the progressive accumulation of low-density lipoprotein cholesterol (LDL-C) and other apolipoprotein (Apo) B containing lipoproteins within the arterial wall. Despite the intensive LDL-C lowering therapy, a substantial residual risk of ASCVD persists and is significantly associated with elevated triglyceride concentrations.

**Objective:**

To review the pathogenesis of triglycerides (TG), triglyceride-rich lipoproteins (TRL) and their remnants as well as their role in the development of atherosclerosis and clinical relevance for assessing residual ASCVD risk.

**Materials and methods:**

A literature search was conducted using keywords relevant to the topic and their combinations. A targeted PubMed search prioritized English-language guidelines, consensus statements, randomized clinical trials, cohort studies, and meta-analyses published between 2019 and 2025, with older landmark publications included where necessary. Reference lists of key articles were also manually screened to identify additional relevant studies.

**Results:**

The literature shows that elevated TG concentrations are associated with a higher risk of ASCVD, regardless of LDL-C. Given that no clear signal indicates which features of TRL give rise to risk of ASCVD, plasma TG levels remain a reasonable surrogate marker for risk assessment. Evidence suggests that the atherogenicity of TRL is driven primarily by the cholesterol carried within TRL and their remnants rather than TG themselves. TRL remnants may be at least as atherogenic as, and potentially more atherogenic than, low-density lipoproteins (LDL) because they can be taken up by intimal macrophages without prior oxidative or structural modification, persist longer within the intima, are larger in size, and carry more cholesterol per particle. They also more effectively promote foam-cell formation and contribute to low-grade inflammation. Given the conflicting results of large-scale randomized clinical trials, it is not yet possible to conclude that lowering TG concentrations alone reduces the ASCVD risk.

**Conclusions:**

Elevated plasma TG concentrations are associated with an increased risk of ASCVD, independent of LDL-C levels. Given the ongoing need to identify the most reliable metric for risk stratification, TG remain a rational surrogate marker for estimating residual ASCVD risk related to TRL and their remnants.

## Abstract (it)

**Įvadas:**

Aterosklerozinė širdies ir kraujagyslių liga (AŠKL) yra lėtinė uždegiminė arterijų liga. Pagrindinė aterosklerozės išsivystymo priežastis – progresuojantis mažo tankio lipoproteinų cholesterolio (MTL-Ch) ir kitų apolipoproteino (Apo) B turinčių lipoproteinų kaupimasis arterijų sienelėse. Nepaisant intensyvaus MTL-Ch kiekį mažinančio gydymo, yra liekamoji AŠKL rizika, kuri reikšmingai siejama su padidėjusia trigliceridų koncentracija.

**Darbo tikslas:**

Apžvelgti trigliceridų (TG), trigliceridų turtingų lipoproteinų (TTL) ir jų remnantų patogenezę, vaidmenį aterosklerozės vystymuisi bei klinikinę reikšmę vertinant liekamąją AŠKL riziką.

**Metodai:**

Atlikta literatūros apžvalga, naudojantis „PubMed“ duomenų baze; papildomai įtrauktos tarptautinės dislipidemijų ir hipertrigliceridemijos gairės. Apibendrinti tyrimai bei apžvalgos, kur nagrinėjama TG, TTL ir jų remnantų įtaka aterosklerozės vystymuisi ir reikšmė liekamajai AŠKL rizikai.

**Rezultatai:**

Literatūros duomenimis, padidėjusi TG koncentracija yra susijusi su didesne AŠKL rizika nepriklausomai nuo MTL-Ch. Kadangi iki galo nėra žinoma, kuri TTL savybė geriausiai prognozuoja AŠKL riziką, plazmos TG koncentracija išlieka racionalus ir praktinis pakaitinis rodiklis su TTL ir jų remnantais susijusiai liekamajai AŠKL rizikai vertinti. Tyrimai rodo, kad TTL aterogeniškumą daugiausia lemia ne patys TG, o TTL ir jų remnantuose esantis cholesterolis. Taip pat manoma, kad TTL remnantai gali būti net labiau aterogeniniai nei mažo tankio lipoproteinai (MTL). Taip yra dėl to, kad jiems nereikia oksidacinių ar struktūrinių pokyčių, kad būtų fagocituoti intimos makrofagų, jie ilgiau užsilieka intimoje, yra didesni ir perneša daugiau cholesterolio molekulių vienoje dalelėje. Be to, jie efektyviau stimuliuoja putliųjų ląstelių formavimąsi ir reikšmingai skatina organizmo uždegiminį atsaką. Esant prieštaringiems didelės apimties klinikinių tyrimų rezultatams, be išlygų teigti, kad vien tik TG koncentracijos sumažinimas mažina AŠKL riziką, kol kas negalima.

**Išvados:**

Padidėjęs trigliceridų kiekis kraujo plazmoje yra reikšmingas AŠKL rizikos veiksnys, nepriklausomai nuo MTL-Ch kiekio. Kai yra poreikis nustatyti patikimiausią šios rizikos vertinimo indeksą, trigliceridai išlieka racionalus rodiklis su TTL ir jų remnantais susijusiai liekamajai AŠKL rizikai vertinti.

## Introduction

*Atherosclerotic Cardiovascular Disease* (ASCVD) is a chronic inflammatory disease of the arteries [1]. According to recent evidence, the principal cause of atherosclerosis development is the progressive retention and accumulation of *Low-Density Lipoprotein Cholesterol* (LDL-C) and other apolipoprotein (Apo) B-containing lipoproteins within the arterial wall [2]. Although the deposition of these lipoproteins is considered the central driver of atherogenesis, the course of atherosclerosis is also substantially modulated by systemic and local inflammation, endothelial dysfunction, and other metabolic processes [3,4]. The most recent *European Society of Cardiology* (ESC) guidelines on the management of dyslipidaemias place the main emphasis on lowering LDL-C concentration – the cornerstone strategy for ASCVD prevention [2,5]. However, even with intensive LDL-C-lowering therapy, achievement of target LDL-C levels, and optimal management of other ASCVD risk factors, a substantial residual risk of ASCVD remains [6–8]. Over the last five years, accumulating evidence has demonstrated that elevated *Triglyceride* (TG) concentrations are associated with a residual ASCVD risk and reflect an increased circulating burden of *Triglyceride-Rich Lipoproteins* (TRLs) and their remnants [7,9]. In this review, TG are considered primarily a biomarker of residual ASCVD risk related to TRLs and their remnants. TRL particles function as carriers of TG and cholesterol, whereas remnant cholesterol represents the directly atherogenic component of these particles. Even in individuals at low or moderate cardiovascular risk, elevated serum TG concentrations are significantly associated with subclinical non-coronary artery atherosclerosis, independently of LDL-C levels [10]. Collectively, these findings support an important role of TRLs and their remnants in atherogenesis. It is considered that the atherogenicity of TRL is driven primarily not by TG themselves, but by the cholesterol content within these particles, commonly referred to as remnant cholesterol [11,12]. Remnant cholesterol can be defined as the cholesterol content of TRL and is calculated as both LDL-C and high-density lipoprotein cholesterol (HDL-C) are subtracted from total plasma cholesterol. Remnant cholesterol is considered particularly relevant because it directly contributes to atherogenesis [11]. Genetic studies have shown that elevated remnant cholesterol concentrations are associated with increased chronic low-grade inflammation, whereas genetically elevated LDL-C concentrations did not demonstrate that type of relationship. These findings suggest that cholesterol carried within TRL significantly modulates the inflammatory component of atherosclerosis, while the contribution of LDL-C to inflammation may be less pronounced [13]. Although TG themselves do not directly participate in the formation of the atherosclerotic plaque, circulating TG levels serve as an important marker for assessing residual atherosclerotic risk [14,15]. Assessment of non–HDL-C and ApoB provides an estimate of the overall burden of atherogenic lipoproteins and their cholesterol content. However, remnant cholesterol offers additional information regarding the risk specifically attributable to TRLs and their remnants, independently of LDL-C concentration [2,16]. Such evaluation is particularly relevant in cases where adverse cardiovascular events occur despite normal LDL-C concentrations, as well as in patients with hypertriglyceridaemia, type 2 diabetes mellitus, metabolic syndrome, or obesity [5]. The aim of this article is to review the role of triglycerides, triglyceride-rich lipoproteins, and remnant cholesterol in the development of atherosclerosis.

## Materials and methods

This article was conducted as a narrative review of the literature on TG, TRL, remnant cholesterol, and their role in the development of atherosclerosis and residual ASCVD risk. A literature search was performed in PubMed using topic-relevant keywords and their combinations, including ‘triglycerides’, ‘triglyceride-rich lipoproteins’, ‘remnant cholesterol’, ‘hypertriglyceridaemia’, ‘residual risk’ and ‘atherosclerosis’. The search prioritized English-language publications, with emphasis on contemporary clinical guidelines, consensus statements, randomized clinical trials, cohort studies, and meta-analyses. Most included publications were published between 2019 and 2025, with older landmark guidelines and foundational studies included where necessary to provide conceptual and mechanistic context. Reference lists of key articles were also manually screened in order to identify additional relevant studies.

## Literature review

### Hypertriglyceridaemia

Elevated circulating TG concentrations are associated with the residual atherosclerotic risk and vascular inflammation [16]. Importantly, this association appears to increase in a dose-dependent manner as TG concentrations rise [10,17]. In the presence of hypertriglyceridaemia, residual atherosclerotic risk increases substantially even among patients with a low or moderate cardiovascular risk and normal LDL-C levels [10]. Accordingly, the following sections review hypertriglyceridaemia, including its etiology, pathogenesis, and clinical relevance to atherosclerosis development.

Hypertriglyceridaemia is a polyetiological condition driven by both genetic factors (e.g., mutations in regions encoding lipoprotein lipase) and secondary causes (obesity, uncontrolled diabetes mellitus, harmful alcohol use, specific commonly used medications, etc.), which lead to an increased synthesis or reduced clearance of TRLs [18]. Hypertriglyceridaemia is most commonly defined as a fasting plasma TG concentration exceeding 1.7 mmol/L [2]. Today, there is no single unified classification of hypertriglyceridaemia as different guidelines and expert panels classify the severity of hypertriglyceridaemia differently and use similar, yet not identical, concentration ranges, as presented in Table 1 [18]. Evidence indicates that hypertriglyceridaemia is an important marker of an increased ASCVD risk when TG concentrations are <10 mmol/L [2,13]. By contrast, studies of patients with extremely high TG concentrations, for example, those with familial chylomicronaemia syndrome, in whom TG levels may exceed 50 mmol/L, have shown a lower risk of ASCVD, which often does not manifest clinically. This can be explained by the fact that the TRL particle size is directly related to the circulating TG levels: at very high TG concentrations (>50 mmol/L), TRLs are too large to enter the arterial intima, and therefore cannot contribute to the formation of atherosclerotic plaque. In contrast, at lower yet elevated TG concentrations (<10 mmol/L), TRL particles and their remnants are sufficiently small to enter the arterial wall and thereby contribute to the development of atherosclerosis [13]. Thus, the ASCVD risk increases when TG concentrations are in the range of 1.7–10 mmol/L, whereas, at levels >10 mmol/L, the risk of pancreatitis becomes the predominant clinical concern. However, in the absence of a clearly defined and universally accepted classification of the severity of hypertriglyceridaemia, the exact numerical threshold, at which the ASCVD risk no longer increases, is not yet established. According to the ESC/EAS guidelines on the management of dyslipidaemias, there is currently no specific TG treatment target for ASCVD prevention. Nevertheless, the guidelines indicate that fasting TG concentrations <1.7 mmol/L are associated with a low ASCVD risk. However, when this threshold is exceeded, the assessment of other cardiovascular risk factors is recommended [2]. In the context of residual risk of atherosclerosis, hypertriglyceridaemia is recognised not as a direct cause of atherosclerosis, but rather as a marker reflecting an increased amount of cholesterol carried by TRLs in plasma [10].

**Table 1:** Classification of hypertriglyceridaemia; based on source [18]

Guidelines/documents	Category	Serum TG concentration mmol/L
National Cholesterol Education Program (NCEP) Expert Panel; 2001 [19]	Normal	<1.7
	Borderline high	1.7 – 2.5
	High	2.6 – 5.6
	Very high	≥5.6
Evaluation and Treatment of Hypertriglyceridemia: An Endocrine Society Clinical Practice Guidelines; 2012 [20]	Normal	<1.7
	Mild	1.7 – 2.3
	Moderate	2.3 – 11.2
	Severe	11.2 – 22.4
	Very severe	>22.4
American Heart Association/American College of Cardiology (AHA/ACC) Guideline on the Management of Blood Cholesterol; 2018 [21]	Moderate	2.0 – 5.6
	Severe	≥5.6
A consensus statement from the European Atherosclerosis Society; 2021 [16]	Optimal	<1.2
	Borderline	1.2 – 1.7
	Moderately elevated	1.7 – 5.7
	Severe	5.7 – 10.0
	Extreme	>10.0

### Mechanisms of atherogenicity of triglyceride-rich lipoproteins

Lipids such as TG and cholesterol are water-insoluble. Therefore, in order to be transported in plasma, they are bound to proteins and form lipoprotein particles. Lipoproteins are classified into six major types: *High-Density Lipoproteins* (HDL), *Low-Density Lipoproteins* (LDL), lipoprotein (a), *Intermediate-Density Lipoproteins* (IDL), TRL – chylomicrons and *Very-Low-Density Lipoproteins* (VLDL) [2]. In this article, we will focus primarily on TRLs, as the cholesterol carried in these lipoproteins and their remnants is thought to play a significant role in atherogenesis.

Accumulating evidence suggests that TRL particles are highly atherogenic and are regarded as being at least as atherogenic as LDL particles [5,12,22,23]. This increased atherogenicity is attributed to several factors. Compared with LDL particles, TRL remnant particles are larger, persist longer in the arterial intima, and do not require oxidative or structural modifications to be phagocytosed by intimal macrophages. Additionally, they carry more cholesterol molecules per particle and promote foam-cell formation more efficiently [5,13,16,22–24]. Moreover, they substantially enhance the inflammatory responses, which are a key component of atherogenesis [13,16,23].

TRL particles and their remnants with a diameter of <70 nm can cross the endothelial barrier via active transcytosis, particularly in the presence of endothelial dysfunction [2,23]. After entering the arterial intima, TRL remnants tend to be retained within the arterial wall because of their particle size and strong electrostatic interactions between their apolipoproteins (ApoB and ApoE) and intimal proteoglycans [23,25]. These interactions are considered to be further enhanced by high levels of ApoC-III expressed on the surface of TRL remnants. In addition, these remnant particles exhibit slower efflux relative to their rate of intimal entry, thus promoting prolonged arterial retention [23].

The plasma residence time of TRL remnant particles is also highly relevant: the longer they persist in the circulation, the more cholesterol molecules they acquire. Each remnant particle can carry up to 7,500 cholesterol molecules, thereby substantially increasing cholesterol delivery to the arterial wall [16].

TRL remnant particles also express ApoE on their surface, which acts as a ligand for the LDL receptor or LRP1 (LDL receptor–related protein 1), on intimal macrophages thereby facilitating receptor-mediated uptake of TRL remnants. Unlike LDL, TRL remnants can be taken up directly by macrophages without requiring chemical or structural modification [16,22,25]. Their accumulation within macrophages promotes foam-cell formation, which is a key process in atherogenesis [23].

Although there is no evidence that TG exert a direct atherogenic effect, studies indicate that elevated TG and remnant cholesterol levels are causally related to systemic low-grade inflammation [16,23,25]. The recent PESA (Progression of Early Subclinical Atherosclerosis) study found a significant association between vascular inflammation and increased TG concentrations when TG values were ≥1.7 mmol/L [16]. TRL remnants promote inflammatory processes through several mechanisms: during LPL-mediated hydrolysis of TG within TRLs and their remnants, oxidized FFA are generated, which promote endothelial inflammation and increase the expression of intracellular adhesion molecules. In addition, TRL remnants increase the production of reactive oxygen species (ROS), tumor necrosis factor-α (TNF-α), and interleukin-1β (IL-1β), leading to impaired endothelium-dependent vasodilation, increased endothelial apoptosis, and oxidative stress [22]. The uptake of TRL remnants by macrophages promotes a pro-inflammatory M1 macrophage phenotype, enhances inflammation, and indirectly contributes to fibrous-cap thinning via an increased metalloproteinase expression [16].

**Figure 1:**
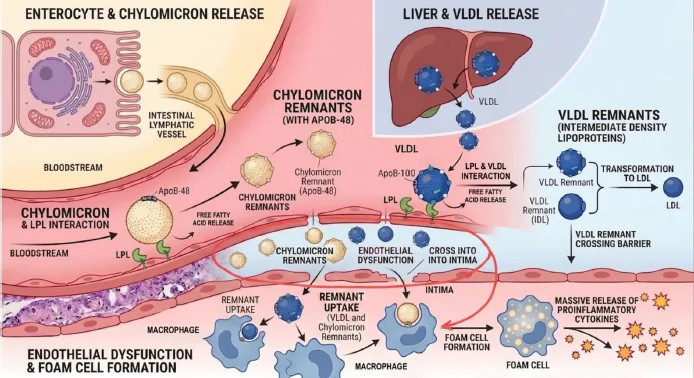
This schematic illustrates the metabolism of chylomicrons and VLDL into TRL remnant particles and their contribution to atherogenesis. Chylomicrons are formed in enterocytes and released into the lymphatic system before entering the bloodstream, whereas VLDL are synthesized and secreted by the liver. In the circulation, both chylomicrons and VLDL undergo lipolysis mediated by LPL, resulting in triglyceride hydrolysis, release of free fatty acids (FFA), and formation of chylomicron and VLDL remnant particles. VLDL remnants may subsequently be converted into LDL. The figure also shows that, particularly in the setting of endothelial dysfunction, remnant particles can penetrate the endothelial barrier and enter the intima, where they are taken up by macrophages, promoting foam cell formation and proinflammatory cytokine release, and thus contributing to atherosclerotic plaque development. Note. Abbreviations used in the Figure: VLDL – very-low density lipoproteins, TRL – triglyceride-rich lipoproteins, LPL – lipoprotein lipase, IDL – intermediate density lipoproteins, FFA – free fatty acids, LDL – low-density lipoproteins.

### Significance of TRLs for the risk of ASCVD

According to the 2025-dated focused update of the ESC/EAS Guidelines for the management of dyslipidaemias, plasma TG concentrations are associated with an increased risk of ASCVD, irrespective of the LDL-C levels [26]. Since there is currently no single marker which would precisely reflect which properties of TRLs best predict the ASCVD risk – and given the ongoing need to identify the most reliable index for risk assessment – plasma TG concentration is considered a rational and practical surrogate marker for estimating the residual ASCVD risk related to TRLs and their remnants [16].

An elevated plasma TG concentration is an important marker of the residual ASCVD risk both in healthy, statin-naïve individuals at a low-to-moderate cardiovascular risk as well as in high-risk patients on statin therapy, particularly those with diabetes mellitus [10,16]. In the study by Raposeiras-Roubin et al., even among individuals denoted by a low or moderate cardiovascular risk, higher serum TG levels were significantly associated with subclinical non-coronary atherosclerosis, independently of the LDL-C concentration [10]. The authors emphasize that TG values are routinely measured, inexpensive, and they are a widely available laboratory parameter that can be applied to ASCVD risk assessment. The study also suggested that hypertriglyceridaemia could serve as a therapeutic target for reducing the residual risk of atherosclerosis and related clinical events [10].

Over the past six years, three large randomized controlled trials – STRENGTH, PROMINENT, and REDUCE-IT – have evaluated the clinical relevance of TG lowering for the risk of *Major Adverse Cardiovascular Events* (MACE) by using different therapeutic strategies. Although the interventions in STRENGTH and PROMINENT reduced TG concentrations, they did not reduce the risk of MACE [27,28]. Whereas, in PROMINENT, reductions in remnant cholesterol were accompanied by increases in LDL-C and apoB, suggesting that the overall atherogenic burden may not have fallen enough to reduce MACE. This supports the idea that the cardiovascular benefit depends more on reducing the total apoB-containing particle burden and the total atherogenic cholesterol burden than on lowering TG alone [28]. By contrast, REDUCE-IT demonstrated that, in patients with established atherosclerotic cardiovascular disease and elevated TG levels, icosapent ethyl significantly reduced the risk of MACE irrespective of the baseline cardiovascular risk, with the greatest absolute benefit observed in the highest-risk patients [29]. Meanwhile, in REDUCE-IT, the cardiovascular benefit of icosapent ethyl was consistent across the baseline triglyceride categories and irrespective of the triglyceride levels attained at 1 year, suggesting that risk reduction was not solely dependent on achieving lower triglyceride concentrations. Moreover, the cardiovascular efficacy of icosapent ethyl is thought to extend beyond triglyceride reduction, by encompassing anti-inflammatory, antioxidative, plaque-stabilizing, and membrane-stabilizing mechanisms [30]. Taken together, these findings suggest that the clinical benefit depends not only on the magnitude of TG lowering, but also on the mechanism by which TG reduction is achieved and on accompanying biochemical changes that contribute to an overall reduction in cardiovascular risk.

The 2019 ESC/EAS Guidelines for the management of dyslipidaemias state that ASCVD risk increases when fasting TG concentrations exceed 1.7 mmol/L. However, pharmacotherapy is recommended only for high-risk patients when TG exceed 2.3 mmol/L after secondary causes have been excluded, or when TG are 1.5–5.6 mmol/L despite the statin therapy [2]. Raposeiras-Roubin et al. note that, in contrast to the current ESC/EAS recommendations, lipid-lowering therapy may be beneficial even in individuals with a low or moderate cardiovascular risk when TG concentration exceeds 1.7 mmol/L [10].

Accordingly, in light of recent evidence, novel therapeutic strategies are being actively developed to reduce circulating TG, TRLs, and their remnants with the aim of achieving an additional cardiovascular benefit [16,31].

### Importance of TG measurement

Although it is not yet fully clear which characteristic of TRL best predicts the ASCVD risk, a substantial body of evidence indicates that the cholesterol carried in TRLs and their remnants is atherogenic and is associated with an increased ASCVD risk [16,32]. However, the assessment of remnant cholesterol in clinical practice is challenging because of technical limitations, a high cost, and lack of standardization. Remnant cholesterol can be assessed either directly or indirectly. Direct measurement requires specialized methods (e.g., nuclear magnetic resonance spectroscopy, ultracentrifugation, or polyacrylamide gel electrophoresis), which are expensive, require method standardization and unified interpretive criteria that are not yet widely implemented in routine practice [16,24,25]. More commonly, remnant cholesterol is estimated indirectly by subtracting LDL-C and HDL-C from the total cholesterol score [16,25]. This approach is simpler, less costly, and does not require specialized equipment. However, its accuracy is limited because it is a derived measure and depends directly on the accuracy of LDL-C determination. When LDL-C is estimated by using the Friedewald equation, a fixed ratio between TG and VLDL cholesterol is assumed [33]. In the clinical practice, this ratio is not constant and may vary within the same individual over time, leading to imprecise remnant cholesterol estimates [33]. This is particularly relevant in patients with more variable TRL composition, such as those with diabetes mellitus or hypertriglyceridaemia. This inaccuracy can be mitigated partially by using the Martin–Hopkins equation, or by measuring LDL-C directly [33]. In addition, there are no widely accepted target concentrations or reference ranges for remnant cholesterol, thus further limiting its routine clinical application.

Elevated TG values correlate closely with increased levels of TRLs and their remnants [25]. Given the limitations of remnant cholesterol measurement and the ongoing need to identify the best predictor of ASCVD risk, measurement of plasma TG is recommended in the clinical practice [16]. TG are considered a practical surrogate marker of residual ASCVD risk related to TRLs and their remnants, and these values can be measured in most laboratories [10,16]. According to the year 2025 updated ESC dyslipidaemia guidelines, the TG concentration should be considered an independent marker of the ASCVD risk even when LDL-C is normal, particularly in patients with metabolic syndrome or diabetes mellitus [26]. In addition, serum TG concentration >1.7 mmol/L has been associated with subclinical atherosclerosis in non-coronary arteries even in the presence of normal LDL-C [10]. Although guideline cut-offs for hypertriglyceridaemia may vary (see Table 1), threshold values have been defined to guide when to initiate treatment and/or intensify lifestyle modification.

## Conclusions

1. Elevated plasma TG concentration is currently regarded as a practical surrogate marker of residual ASCVD risk related to TRLs and their remnants.
2. The atherogenicity of triglyceride-rich lipoproteins is driven primarily not by TG themselves, but by the cholesterol content within these particles. TRL remnants are thought to be at least as atherogenic as, and potentially more atherogenic than, LDL particles.
3. Given the discordant results of large-scale clinical trials, it is not yet possible to state uniformly that TG reduction alone lowers the ASCVD risk. Clinical benefit depends not only on the degree of TG reduction but also on the mechanisms by which this reduction is achieved.

## References

[1] Yanai H, Adachi H, Hakoshima M, Katsuyama H (2022). Atherogenic lipoproteins for the statin residual cardiovascular disease risk. *Int J Mol Sci*.

[2] Mach F, Baigent C, Catapano AL (2020). 2019 ESC/EAS Guidelines for the management of dyslipidaemias: lipid modification to reduce cardiovascular risk. *Eur Heart J*.

[3] Jebari-Benslaiman S, Galicia-García U, Larrea-Sebal A (2022). Pathophysiology of Atherosclerosis. *Int J Mol Sci*.

[4] Hansen SEJ, Madsen CM, Varbo A, Nordestgaard BG (2019). Low-Grade inflammation in the association between mild-to-moderate hypertriglyceridemia and risk of acute pancreatitis: a study of more than 115000 individuals from the general population. *Clin Chem*.

[5] Pintó X, Fanlo M, Esteve V (2023). Colesterol remanente, riesgo vascular y prevención de la arteriosclerosis. *Clín Investig Arterioscler*.

[6] Shi F, Dou J, Zhang X (2025). Advancements in research to mitigate residual risk of atherosclerotic cardiovascular disease. *Eur J Med Res*.

[7] Farnier M, Zeller M, Masson D, Cottin Y (2021). Triglycerides and risk of atherosclerotic cardiovascular disease: An update. *Arch Cardiovasc Dis*.

[8] Zheng WC, Chan W, Dart A, Shaw JA (2024). Novel therapeutic targets and emerging treatments for atherosclerotic cardiovascular disease. *Eur Heart J Cardiovasc Pharmacother*.

[9] Quispe R, Sweeney T, Varma B, Agarwala A, Michos ED (2022). Recent Updates in Hypertriglyceridemia Management for Cardiovascular Disease Prevention. *Curr Atheroscler Rep*.

[10] Raposeiras-Roubin S, Rosselló X, Oliva B (2021). Triglycerides and residual atherosclerotic risk. *J Am Coll Cardiol*.

[11] Drexel H, Mader A, Larcher B (2025). Remnant cholesterol and long-term incidence of death in coronary artery disease patients. *Atherosclerosis*.

[12] Li X, Li ZF, Wu NQ (2025). Remnant Cholesterol and Residual Risk of Atherosclerotic Cardiovascular Disease. *Rev Cardiovasc Med*.

[13] Nordestgaard BG, Varbo A (2014). Triglycerides and cardiovascular disease. *The Lancet*.

[14] Heo JH, Jo SH (2023). Triglyceride-rich lipoproteins and remnant cholesterol in cardiovascular disease. *J Korean Med Sci*.

[15] Aaseth E, Halvorsen S, Helseth R, Gravning J (2025). Remnant cholesterol, plasma triglycerides, and risk of cardiovascular disease events in young adults: a prospective cohort study. *Eur J Prev Cardiol*.

[16] Ginsberg HN, Packard CJ, Chapman MJ (2021). Triglyceride-rich lipoproteins and their remnants: metabolic insights, role in atherosclerotic cardiovascular disease, and emerging therapeutic strategies—a consensus statement from the European Atherosclerosis Society. *Eur Heart J*.

[17] Koo BK, Park S, Han KD, Moon MK (2021). Hypertriglyceridemia is an independent risk factor for cardiovascular diseases in Korean adults aged 30–49 years: a nationwide population-based study. *J Lipid Atheroscler*.

[18] Simha V (2020). Management of hypertriglyceridemia. *BMJ*.

[19] Expert Panel On Detection, Evaluation, And Treatment Of High Blood Cholesterol In Adults (2001). Executive summary of the third report of the National Cholesterol Education Program (NCEP) Expert Panel on detection, evaluation, and treatment of high blood cholesterol in adults (Adult Treatment Panel III). *JAMA*.

[20] Berglund L, Brunzell JD, Goldberg AC (2012). Evaluation and treatment of hypertriglyceridemia: an Endocrine Society clinical practice guideline. *J Clin Endocrinol Metab*.

[21] Grundy SM, Stone NJ, Bailey AL (2019). 2018 AHA/ACC/AACVPR/AAPA/ABC/ACPM/ADA/AGS/APhA/ASPC/NLA/PCNA Guideline on the Management of Blood Cholesterol: A Report of the American College of Cardiology/American Heart Association Task Force on Clinical Practice Guidelines. *Circulation*.

[22] Rosenson RS, Shaik A, Song W (2021). New therapies for lowering triglyceride-rich lipoproteins. *J Am Coll Cardiol*.

[23] Salinas CAA, Chapman MJ (2020). Remnant lipoproteins: are they equal to or more atherogenic than LDL?. *Curr Opin Lipidol*.

[24] Baratta F, Cocomello N, Coronati M (2023). Cholesterol remnants, triglyceride-rich lipoproteins and cardiovascular risk. *Int J Mol Sci*.

[25] Nordestgaard BG (2016). Triglyceride-rich lipoproteins and atherosclerotic cardiovascular disease: new insights from epidemiology, genetics, and biology. *Circ Res*.

[26] Mach F, Koskinas KC, Roeters Van Lennep JE (2025). 2025 Focused Update of the 2019 ESC/EAS Guidelines for the management of dyslipidaemias. *Eur Heart J*.

[27] Nicholls SJ, Lincoff AM, Garcia M (2020). Effect of high-dose omega-3 fatty acids vs corn oil on major adverse cardiovascular events in patients at high cardiovascular risk: the STRENGTH randomized clinical trial. *JAMA*.

[28] Doi T, Langsted A, Nordestgaard BG (2024). Remnant cholesterol, LDL cholesterol, and apoB absolute mass changes explain results of the PROMINENT trial. *Atherosclerosis*.

[29] Burger PM, Bhatt DL, Dorresteijn JAN (2024). Effects of icosapent ethyl according to baseline residual risk in patients with atherosclerotic cardiovascular disease: results from REDUCE-IT. *Eur Heart J Cardiovasc Pharmacother*.

[30] Bhatt DL, Steg PG, Miller M (2019). Cardiovascular Risk Reduction with Icosapent Ethyl for Hypertriglyceridemia. *N Engl J Med*.

[31] Laufs U, Parhofer KG, Ginsberg HN, Hegele RA (2020). Clinical review on triglycerides. *Eur Heart J*.

[32] Delialis D, Georgiopoulos G, Aivalioti E (2023). Remnant cholesterol in atherosclerotic cardiovascular disease: A systematic review and meta-analysis. *Hellenic J Cardiol*.

[33] Varbo A, Nordestgaard BG (2021). Directly measured vs. calculated remnant cholesterol identifies additional overlooked individuals in the general population at higher risk of myocardial infarction. *Eur Heart J*.

